# Improving the Quality of Web Surveys: The Checklist for Reporting Results of Internet E-Surveys (CHERRIES)

**DOI:** 10.2196/jmir.6.3.e34

**Published:** 2004-09-29

**Authors:** Gunther Eysenbach

## Abstract

Analogous to checklists of recommendations such as the CONSORT statement (for randomized trials), or the QUORUM statement (for systematic reviews), which are designed to ensure the quality of reports in the medical literature, a checklist of recommendations for authors is being presented by the Journal of Medical Internet Research (JMIR) in an effort to ensure complete descriptions of Web-based surveys. Papers on Web-based surveys reported according to the CHERRIES statement will give readers a better understanding of the sample (self-)selection and its possible differences from a “representative” sample. It is hoped that author adherence to the checklist will increase the usefulness of such reports.

## Introduction

The Internet is increasingly used for online surveys and Web-based research. In this issue of the *Journal of Medical Internet Research* we publish two methodological studies exploring the characteristics of Web-based surveys compared to mail-based surveys [1,2]. In previous issues we have published Web-based research such as a survey among physicians conducted on a Web site [3].

As explained in an accompanying editorial [4] as well as in a previous review [5], such surveys can be subject to considerable bias. In particular, bias can result from 1) the non-representative nature of the Internet population and 2) the self-selection of participants (volunteer effect). Often online surveys have a very low response rate (if the number of visitors is used as denominator). Thus, considerable debate ensues about the validity of online surveys. The editor and peer reviewers of this journal are frequently faced with the question of whether to accept for publication studies reporting results from Web surveys (or email surveys). There is no easy answer to this question. Often it “just depends”. It depends on the reasons for the survey in the first place, its execution, and the authors' conclusions. Conclusions drawn from a convenience sample are limited and need to be qualified in the discussion section of a paper. On the other hand, we will not, as many other journals do, routinely reject reports of Web surveys, even surveys with very small response rates, which are typical of electronic surveys, but decide on a case-by-case basis whether the conclusions drawn from a Web survey are valid and useful for readers. Web surveys may be of some use in generating hypotheses which need to be confirmed in a more controlled environment; or they may be used to pilot test a questionnaire or to conduct a Web-based experiment. Statistical methods such as propensity scores may be used to adjust results [4]. Again, it all depends on why and how the survey was done.

Every biased sample is an unbiased sample of another target population, and it is sometimes just a question of defining for which subset of a population the conclusions drawn are assumed to be valid. For example, the polling results on the CNN Web site are certainly highly biased and not representative for the US population. But it is legitimate to assume that they are “representative” for visitors to the CNN Web site who choose to participate in the online survey.

This illustrates the critical importance of carefully describing how and in what context the survey was done, and how the sample, which chose to reply, is constituted and might differ from a representative population-based sample. For example, it is very important to describe the content and nature of the Web site where the survey was posted in order to get an idea of the people who filled in the questionnaire (ie, to characterize the population of respondents). A survey on an anti-vaccination Web site run by concerned parents will have a different visitor structure than, for example, a vaccination clinic site. It is also important to describe in sufficient detail exactly how the questionnaire was administered. For example, was it mandatory that every visitor who wanted to enter the Web site fill it in, or were any other incentives offered? A mandatory survey is likely to reduce a volunteer bias.

Analogous to checklists of recommendations such as the CONSORT statement (for randomized trials), or the QUORUM statement (for systematic reviews), which are designed to ensure the quality of reports in the medical literature, a checklist of recommendations for authors is being presented by JMIR in an effort to ensure complete descriptions of e-survey methodology. Papers reported according to the CHERRIES statement will give peer reviewers and readers a better understanding of the sample selection and its possible differences from a “representative” sample.

## The CHERRIES Checklist

We define an e-survey as an electronic questionnaire administered on the Internet or an Intranet. Although many of the CHERRIES items are also valid for surveys administered via e-mail, the checklist focuses on Web-based surveys.

While most items on the checklist are self-explanatory, a few comments about the “response rate” are in order. In traditional surveys investigators usually report a response rate (number of people presented with a questionnaire divided by the number of people who completed the questionnaire) to allow some estimation of the degree of representativeness and bias. Surveys with response rates lower than 70% or so (an arbitrary cut-off point!) are usually viewed with skepticism.

In online surveys, there is no single response rate. Rather, there are multiple potential methods for calculating a response rate, depending on what are chosen as the numerator and denominator. As there is no standard methodology, we suggest avoiding the term “response rate” and have defined how, at least in this journal, response metrics such as, what we call, the view rate, participation rate and completion rate should be calculated.

A common concern for online surveys is that a single user fills in the same questionnaire multiple times. Some users like to go back to the survey and experiment with the results of their modified entries. Multiple methods are available to prevent this or at least to minimize the chance of this happening (eg, cookies or log-file/IP address analysis).

Investigators should also state whether the completion or internal consistency of certain (or all) items was enforced using Javascript (ie, displaying an alert before the questionnaire can be submitted) or server-side techniques (ie, after submission displaying the questionnaire and highlighting mandatory but unanswered items or items answered inconsistently).

The hope is that the CHERRIES checklist provides a useful starting point for investigators reporting results of Web surveys. The editor and peer reviewers of this journal ask authors to ensure that they report the methodology fully and according to the CHERRIES checklist before submitting manuscripts.

**Table 1:** Checklist for Reporting Results of Internet E-Surveys (CHERRIES)

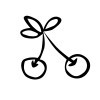	**Checklist for Reporting Results of Internet E-Surveys (CHERRIES)**	
*** Item Category***	***Checklist Item***	***Explanation***
**Design**		
	Describe survey design	Describe target population, sample frame. Is the sample a convenience sample? (In “open” surveys this is most likely.)
**IRB (Institutional Review Board) approval and informed consent process**		
	IRB approval	Mention whether the study has been approved by an IRB.
	Informed consent	Describe the informed consent process. Where were the participants told the length of time of the survey, which data were stored and where and for how long, who the investigator was, and the purpose of the study?
	Data protection	If any personal information was collected or stored, describe what mechanisms were used to protect unauthorized access.
**Development and pre-testing**		
	Development and testing	State how the survey was developed, including whether the usability and technical functionality of the electronic questionnaire had been tested before fielding the questionnaire.
**Recruitment process and description of the sample having access to the questionnaire**		
	Open survey versus closed survey	An “open survey” is a survey open for each visitor of a site, while a closed survey is only open to a sample which the investigator knows (password-protected survey).
	Contact mode	Indicate whether or not the initial contact with the potential participants was made on the Internet. (Investigators may also send out questionnaires by mail and allow for Web-based data entry.)
	Advertising the survey	How/where was the survey announced or advertised? Some examples are offline media (newspapers), or online (mailing lists – If yes, which ones?) or banner ads (Where were these banner ads posted and what did they look like?). It is important to know the wording of the announcement as it will heavily influence who chooses to participate. Ideally the survey announcement should be published as an appendix.
**Survey administration**		
	Web/E-mail	State the type of e-survey (eg, one posted on a Web site, or one sent out through e-mail). If it is an e-mail survey, were the responses entered manually into a database, or was there an automatic method for capturing responses?
	Context	Describe the Web site (for mailing list/newsgroup) in which the survey was posted. What is the Web site about, who is visiting it, what are visitors normally looking for? Discuss to what degree the content of the Web site could pre-select the sample or influence the results. For example, a survey about vaccination on a anti-immunization Web site will have different results from a Web survey conducted on a government Web site
	Mandatory/voluntary	Was it a mandatory survey to be filled in by every visitor who wanted to enter the Web site, or was it a voluntary survey?
	Incentives	Were any incentives offered (eg, monetary, prizes, or non-monetary incentives such as an offer to provide the survey results)?
	Time/Date	In what timeframe were the data collected?
	Randomization of items or questionnaires	To prevent biases items can be randomized or alternated.
	Adaptive questioning	Use adaptive questioning (certain items, or only conditionally displayed based on responses to other items) to reduce number and complexity of the questions.
	Number of Items	What was the number of questionnaire items per page? The number of items is an important factor for the completion rate.
	Number of screens (pages)	Over how many pages was the questionnaire distributed? The number of items is an important factor for the completion rate.
	Completeness check	It is technically possible to do consistency or completeness checks before the questionnaire is submitted. Was this done, and if “yes”, how (usually JAVAScript)? An alternative is to check for completeness after the questionnaire has been submitted (and highlight mandatory items). If this has been done, it should be reported. All items should provide a non-response option such as “not applicable” or “rather not say”, and selection of one response option should be enforced.
	Review step	State whether respondents were able to review and change their answers (eg, through a Back button or a Review step which displays a summary of the responses and asks the respondents if they are correct).
**Response rates**		
	Unique site visitor	If you provide view rates or participation rates, you need to define how you determined a unique visitor. There are different techniques available, based on IP addresses or cookies or both.
	View rate (Ratio of unique survey visitors/unique site visitors)	Requires counting unique visitors to the first page of the survey, divided by the number of unique site visitors (not page views!). It is not unusual to have view rates of less than 0.1 % if the survey is voluntary.
	Participation rate (Ratio of unique visitors who agreed to participate/unique first survey page visitors)	Count the unique number of people who filled in the first survey page (or agreed to participate, for example by checking a checkbox), divided by visitors who visit the first page of the survey (or the informed consents page, if present). This can also be called “recruitment” rate.
	Completion rate (Ratio of users who finished the survey/users who agreed to participate)	The number of people submitting the last questionnaire page, divided by the number of people who agreed to participate (or submitted the first survey page). This is only relevant if there is a separate “informed consent” page or if the survey goes over several pages. This is a measure for attrition. Note that “completion” can involve leaving questionnaire items blank. This is not a measure for how completely questionnaires were filled in. (If you need a measure for this, use the word “completeness rate”.)
**Preventing multiple entries from the same individual**		
	Cookies used	Indicate whether cookies were used to assign a unique user identifier to each client computer. If so, mention the page on which the cookie was set and read, and how long the cookie was valid. Were duplicate entries avoided by preventing users access to the survey twice; or were duplicate database entries having the same user ID eliminated before analysis? In the latter case, which entries were kept for analysis (eg, the first entry or the most recent)?
	IP check	Indicate whether the IP address of the client computer was used to identify potential duplicate entries from the same user. If so, mention the period of time for which no two entries from the same IP address were allowed (eg, 24 hours). Were duplicate entries avoided by preventing users with the same IP address access to the survey twice; or were duplicate database entries having the same IP address within a given period of time eliminated before analysis? If the latter, which entries were kept for analysis (eg, the first entry or the most recent)?
	Log file analysis	Indicate whether other techniques to analyze the log file for identification of multiple entries were used. If so, please describe.
	Registration	In “closed” (non-open) surveys, users need to login first and it is easier to prevent duplicate entries from the same user. Describe how this was done. For example, was the survey never displayed a second time once the user had filled it in, or was the username stored together with the survey results and later eliminated? If the latter, which entries were kept for analysis (eg, the first entry or the most recent)?
**Analysis**		
	Handling of incomplete questionnaires	Were only completed questionnaires analyzed? Were questionnaires which terminated early (where, for example, users did not go through all questionnaire pages) also analyzed?
	Questionnaires submitted with an atypical timestamp	Some investigators may measure the time people needed to fill in a questionnaire and exclude questionnaires that were submitted too soon. Specify the timeframe that was used as a cut-off point, and describe how this point was determined.
	Statistical correction	Indicate whether any methods such as weighting of items or propensity scores have been used to adjust for the non-representative sample; if so, please describe the methods.
