# Correction: Improving the Quality of Web Surveys: the Checklist for Reporting Results of Internet E-Surveys (CHERRIES)

**DOI:** 10.2196/jmir.2042

**Published:** 2012-01-04

**Authors:** Gunther Eysenbach

**Affiliations:** ^1^University Health NetworkCentre for Global eHealth Innovation & Techna InstituteToronto, ONCanada; ^2^Institute for Health Policy, Management, and EvaluationUniversity of TorontoToronto, ONCanada; ^3^JMIR Publications Inc.Toronto, ONCanada

An error in the CHERRIES statement has been corrected (J Med Internet Res 2004;6[3]:e34). In the original paper, in table 1, under the recommendations on how response rates (view rate, participation rate, and completion rate) should be calculated, denominators and numerators were flipped. The view rate should be the ratio of unique survey visitors divided by unique site visitors. The participation rate should be the ratio of those who agreed to participate divided by unique first survey page visitors. The completion rate is the ratio of the number of people who finished the survey divided by those who agreed to participate. The corrections have been made in the table in both columns. A corrected version has been submitted to PubMed Central, but incorrect versions may exist on other sites.

